# Role of n-3 Polyunsaturated Fatty Acids in Ameliorating the Obesity-Induced Metabolic Syndrome in Animal Models and Humans

**DOI:** 10.3390/ijms17101689

**Published:** 2016-10-09

**Authors:** Chao-Wei Huang, Yi-Shan Chien, Yu-Jen Chen, Kolapo M. Ajuwon, Harry M. Mersmann, Shih-Torng Ding

**Affiliations:** 1 Department of Animal Science and Technology, National Taiwan University, Taipei 106, Taiwan; d98626004@ntu.edu.tw (C.-W.H.); b02606004@ntu.edu.tw (Y.-S.C.); mersmann@msn.com (H.M.M.); 2 Institute of Biotechnology, National Taiwan University, Taipei 106, Taiwan; d01642005@ntu.edu.tw; 3 Department of Animal Science, Purdue University, West Lafayette, IN 47907-2054, USA; kajuwon@purdue.edu

**Keywords:** n-3 polyunsaturated fatty acids, docosahexaenoic acid, eicosapentaenoic acid, lipid metabolism, energy expenditure

## Abstract

The incidence of obesity and its comorbidities, such as insulin resistance and type II diabetes, are increasing dramatically, perhaps caused by the change in the fatty acid composition of common human diets. Adipose tissue plays a role as the major energy reservoir in the body. An excess of adipose mass accumulation caused by chronic positive energy balance results in obesity. The n-3 polyunsaturated fatty acids (n-3 PUFA), DHA (docosahexaenoic acid) and EPA (eicosapentaenoic acid) exert numerous beneficial effects to maintain physiological homeostasis. In the current review, the physiology of n-3 PUFA effects in the body is delineated from studies conducted in both human and animal experiments. Although mechanistic studies in human are limited, numerous studies conducted in animals and models in vitro provide potential molecular mechanisms of the effects of these fatty acids. Three aspects of n-3 PUFA in adipocyte regulation are discussed: (1) lipid metabolism, including adipocyte differentiation, lipolysis and lipogenesis; (2) energy expenditure, such as mitochondrial and peroxisomal fatty acid β-oxidation; and (3) inflammation, including adipokines and specialized pro-resolving lipid mediators. Additionally, the mechanisms by which n-3 PUFA regulate gene expression are highlighted. The beneficial effects of n-3 PUFA may help to reduce the incidence of obesity and its comorbidities.

## 1. Introduction

Obesity has become a health problem worldwide [1], and it leads to increase activation of inflammatory signaling in both immune and non-immune cells [2]. Consequently, obesity and its comorbidities, characterized by hyperglycemia, dyslipidemia, abdominal obesity, low-grade systemic inflammation, hypertension, elevated plasma triacylglycerol (TAG) and cholesterol, may lead to the onset of metabolic syndrome, a serious threat to the health of the population [1,3]. The major cause of obesity is the chronic excessive energy intake from calorie-dense foods, such as high fat foods that contain greater caloric content than the carbohydrate or protein constituents coupled with decreased energy expenditure [4,5]. In addition, the increased ratio of n-6 to n-3 polyunsaturated fatty acid (PUFA) in modern foods probably plays a role, as well. The ratio of n-6 to n-3 PUFA was probably equal in early human diets, but as vegetable oils (soybean, corn, sunflower, safflower and cottonseed oils), abundant in linoleic acid (LA; C18:2 n-6), became popularized for cooking, there was a significant increase in n-6 PUFA intake, leading to distortion in this ratio. The n-6/n-3 ratio in the typical Western diet is now close to 20:1, instead of 1:1 [5,6]. Furthermore, high intake of n-6 PUFA during the prenatal period is associated with increased adiposity in the offspring [7]. The ratio of n-6/n-3 affects energy balance dramatically, perhaps because these fatty acids have the highest rates of mobilization into the plasma [8] and also because n-3 PUFA may alter the release of lipid mediators or adipokines from adipose tissues. Among the n-3 PUFA, eicosapentaenoic acid (EPA; C20:5 n-3), docosapentaenoic acid (DPA; C22:5n-3) and docosahexaenoic acid (DHA; C22:6 n-3) are highly beneficial, and EPA is mobilized and metabolized more rapidly than the others [9].

Adipose tissue has evolved as an energy-buffering organ, which stores excessive fatty acids as TAG under conditions of positive energy balance and releases fatty acids during negative energy balance. The half-life of adipose tissue lipids is between six and nine months [10]. Thus, in weight-stable individuals, the fatty acid composition of adipose tissue can be considered as an indicator of dietary fatty acid composition [11]. When adipose tissue expansion cannot accommodate the availability of excess fatty acids, the excess is deposited in non-adipose organs, and the toxicity of fatty acids has been attributed to the inability of cells to incorporate them into neutral lipid droplets. The ectopic TAG deposition is in undesirable sites, such as the liver, heart and skeletal muscle, and accumulation of TAG in the liver leads to steatosis [12,13,14,15]. As adipose tissue expands, macrophages infiltrate the tissue, leading to increased inflammation signaling and leukocyte accumulation [2].

The PUFA intake by Yup’ik Eskimo people is more than 30-times greater than the current mean intake of the general U.S. population (4.1 ± 0.5 g/day versus 0.05 g/day in men; 2.8 ± 0.3 g/day versus 0.09 g/day in women). The Eskimos have low plasma TAG and very low density lipoprotein (VLDL) [16], as well as a decreased risk of coronary atherosclerosis [17]. Several world governments and health organizations have established recommendations for n-3 PUFA intake because of their beneficial effects [18]. In humans, the conversion from alpha-linolenic acid (ALA; C18:3 n-3) to EPA is low (~8%) with even lower conversion to DHA (<0.1%) [19,20]. In rodents after a 5-min infusion of ALA, there is little (liver) or no (brain) newly-synthesized EPA or DHA [21,22]. Furthermore, unlike EPA and DHA, ALA has no effect on adiposity and body weight loss [23]. These data suggest that EPA/DHA are a better option for increasing n-3 fatty acid intake than ALA and should be consumed directly. The EPA and DHA can be stored in lipid fractions, such as phospholipids, including in red blood cells and plasma phospholipids. n-3 PUFA are not only essential nutrients, but also beneficial mediators for preventing metabolic syndrome.

However, perhaps the best remedy for obesity, metabolic syndrome or non-alcoholic fatty liver disease prevention is caloric restriction and/or increased energy expenditure. TAG is a marker for total fats in circulation and metabolic syndrome, which is associated with increased plasma TAG [24]. Thus, to lower the plasma TAG oxidation or storage in tissues, n-3 PUFA supplementation appears to be a viable treatment option. Decreased TAG synthesis via inhibited de novo lipogenesis or re-esterification of fatty acids within tissues leads to lower delivery of fatty acids to tissues and a decreased lipotoxicity in organs. The origin of fatty acids deposited in fat tissues can be from dietary sources, de novo lipid synthesis or both. In humans, liver is the major metabolic organ for anabolic and catabolic processes, including the regulation of fat metabolism, e.g., cholesterol synthesis, de novo lipogenesis and the synthesis of apolipoprotein B100. In addition, dietary fats regulates glycolysis, de novo lipogenesis, fatty acid elongation, desaturation and oxidation to control hepatic carbohydrate and lipid metabolism [25]. Dietary DHA is esterified into chylomicrons during the process of gastrointestinal absorption and is also packaged into VLDL by the liver [26]. The contribution of hepatic de novo lipid synthesis (DNL) to new TAG is rather low compared with the intake of relatively greater amounts of fats from the diet [27].

**Figure 1:**
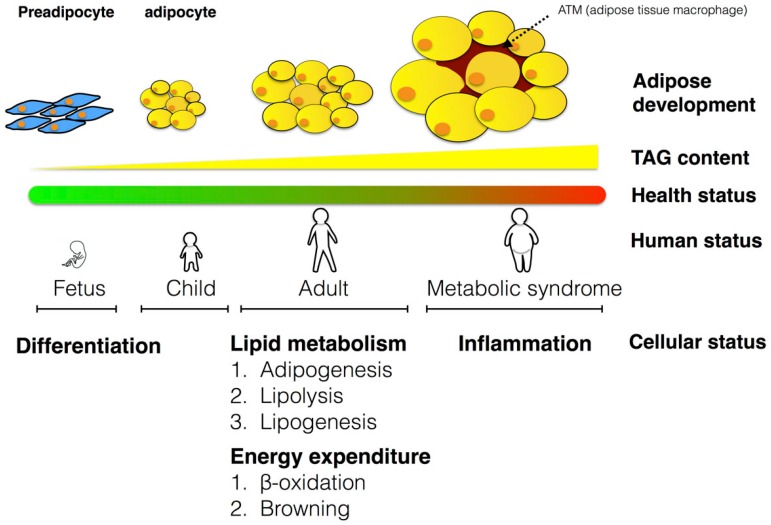
Schematic representation of the physiological changes of adipocytes, including adipose development in different periods of life, and the major mechanisms involved in energy homeostasis. The adipose development proceeds from preadipocytes to adipocytes. The health status from green to red, indicates healthy to unhealthy status. The developmental stages in humans are divided into four major categories, fetal stage, infant stage, adult stage and obese adult stage, to reflect the changing status of adipose tissues throughout life. The cellular status reflects the molecular mechanisms involved in human adipose tissue development. TAG: triacylglycerol.

This review highlights the effect of n-3 PUFA from humans and animal models on molecular mechanisms. Knowledge of the mechanisms involved in the effects of n-3 PUFA on humans is limited, and data from animal models and in vitro studies have been included to ferret out the mechanisms involved. Herein, we focused on three aspects of the effects of n-3 PUFA in adipose tissue: effects on lipid metabolism, energy expenditure and inflammation. The review will also address the specific effects of n-3 PUFA to ameliorate obesity-induced metabolic syndrome (Figure 1). Although this review is focused on the effects and metabolism of n-3 PUFA in adipose tissue, effects on the liver and hepatokine production will also be discussed.

## 2. Methodology of Reference Selection

The review was inspired by previous reviews to identify the beneficial effects of DHA in adipose tissue [27,28,29,30]. The literature search strategy focused on articles published in peer-reviewed and English-language journals between the years 1951 and 2016. The databases used were PubMed and Google Scholar. Paper selection focused on the fields of nutrition, metabolism and molecular mechanisms. Keywords used in each electronic database search included obesity, diabetes, adipose tissue, fatty acids, fish oil, n-3 polyunsaturated fatty acid (n-3 PUFA), animal models, lipid metabolism, inflammation, etc.

## 3. The Role of Adipose Tissues in Metabolism

Adipose tissue depots are of two types, white adipose tissue (WAT) and brown adipose tissue (BAT) [31]. The basic role of adipose tissue is for the storage of excess neutral lipids and dissipation of stored energy. Knowledge regarding regulation of WAT and BAT lipid metabolism could yield strategies to prevent and manage obesity and the related metabolic syndrome.

WAT is a highly plastic and dynamic tissue and accounts for less than 5% of body weight in very lean individuals and over 60% in very obese individuals [32]. Approximately 10% of adipocytes are renewed each year, indicating the dynamic state of adipocytes [33]. Excessive accumulation of WAT is determined by its lipid turnover, which includes synthesis and degradation of TAG and fatty acid oxidation in adipocytes. Excessive accumulation of WAT leads to obesity, which may be caused by an increase in adipocyte numbers (hyperplasia) and/or adipocyte size (hypertrophy) [34]. Altered lipolysis is probably a consequence of increased fat mass rather than a factor preceding the development of being overweight/obesity because increased lipolysis during the development of obesity would be expected to decrease fat mass. Adipose tissue mass is also determined by the rate of lipid turnover, and this includes the synthesis and degradation of TAG and oxidation in adipocytes. Hence, adipose tissue lipolysis is negatively correlated with the current body mass index (BMI) [35]. Adipose tissue lipolytic activity increases after a 5% diet-induced weight loss, and 5% weight loss improves pancreatic β-cell function and insulin sensitivity in muscles. Furthermore, a 5% weight loss is enough to increase insulin-stimulated glucose uptake by ~25% in obese people with insulin resistance [36]. The reduction in adipogenic gene expression with obesity suggests that adipocytes from obese mice or humans have a plateaued lipogenic capacity, leading to a reduced rate of TAG accumulation resulting in ectopic fat accumulation in the liver and other organs [27].

Most mammals react to cold through shivering or non-shivering thermogenesis to increase heat production. Non-shivering thermogenesis is active mainly in BAT [37]. BAT, as well as beige adipocytes (derived from white adipose tissues with many characteristics of brown adipocytes), largely present in infants or newborn mammals play a central role in regulating energy homeostasis via thermogenesis that is regulated by mitochondrial uncoupling protein 1 (UCP1) [38]. In most eukaryotic cells, oxidative phosphorylation (OXPHOS) is a process by which adenosine triphosphate (ATP) is formed as electrons traverse the electron transport chain. However, BAT expresses unique UCP1 proteins, which oxidize fuel substrates and uncouple the respiratory chain from the production of ATP to generate heat [39]. Furthermore, very recent data obtained by positron emission tomography (PET) indicate that active BAT is also present in adult humans [40,41]. BAT-mediated thermogenesis is mainly activated by the hypothalamus via the sympathetic nervous system [42].

Recently, a group of brown-like adipocytes in the classical WAT depot called “brite (brown in-white) or beige” adipocytes have been identified. Even though the UCP1 level is lower in these cells than in the classic brown adipocytes, thermogenesis can also be induced by appropriate stimulation, such as environmental conditions (e.g., the cold) or nutrients (e.g., capsaicin, curcumin, fish oils) [43,44,45]. Furthermore, the mitochondrial respiration and energy expenditure can be similar to that of brown adipocytes [38,46].

## 4. Metabolism of Dietary n-3 Polyunsaturated Fatty Acids (n-3 PUFA)

**Figure 2:**
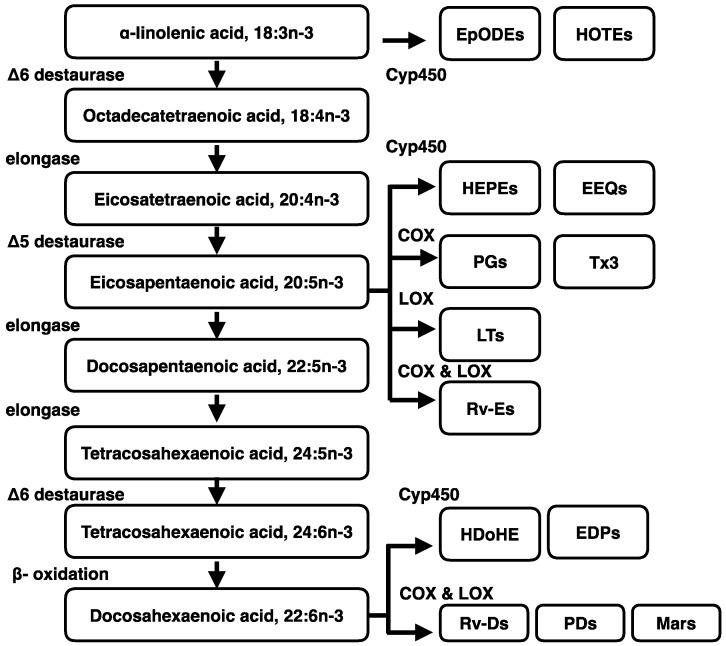
Biosynthesis of n-3 PUFA and its metabolites. EpODEs, epoxyoctadecadienoic acid; EPD, epoxydocosapentaenoic acid; HOTEs, hydroxyoctadecatrienoic acid; HEPEs, hydroxyeicosapentaenoic acids; Tx3, thromboxanes; EEQs, epoxyeicosatetraenoic acid; PGs, prostaglandins; LTs, leukotrienes; COX, cyclooxygenase; LOX, lipoxygenase; Rv-Es, resolvin-Es; HDoHE, hydroxydocosahexaenoic acid; Rv-Ds, resolvin-Ds; PDs, protectin; Mars, maresins.

n-3 and n-6 are the two major classes of PUFA in the diet and are required for optimal human health. n-3 and n-6 PUFA contain the first cis double bond between the third and fourth carbon atoms (n-3) and the sixth and seventh carbon atoms (n-6) from the methyl end of the fatty acids, respectively [47]. Linoleic acid (LA; C18:2 n-6) and ALA (C18:3 n-3) are the predominant plant-derived PUFA. Each of these PUFA is the parent fatty acid for the synthesis of the n-6 and n-3 series of fatty acids in mammals [48]. Although sufficient PUFA cannot be synthesized de novo, it has become apparent that adipose tissues can serve as a reservoir for PUFA [49]. In rodents and humans, following dietary intake of ALA, a significant proportion is directed to adipose tissues, and ALA is converted to DHA in the liver and exported to the blood [50]. Metabolism of ALA includes β-oxidation, extensive carbon recycling, elongation and desaturation [51]. There is 65%–80% of ALA shunted toward β-oxidation [52]. Clinical studies demonstrate that endogenous conversion of ALA to DHA is limited and insufficient to meet the DHA requirement for tissue phospholipids [20]. The n-3 fatty acids, EPA and DHA can only be marginally synthesized from ALA. About 0.3%–7% of ALA is converted to EPA and less than 0.01% to DHA [53,54]. The conversion rate of ALA into DHA is about 1% in infants, and the rate is even lower in adults [55]. The reactions of the desaturation and elongation of LA or ALA share the same enzyme complexes [23]. Compared to EPA (C20:5 n-3), DHA (C22:6 n-3) is more abundant in most tissues, but in contrast to EPA. ALA is not efficiently converted to DHA [56]. For example, when mouse WAT and BAT cells are treated with deuterated ALA (d5-18:3 n-3), undifferentiated BAT cells accumulate EPA and DPA with only a small amount of DHA synthesis. In contrast, differentiated BAT cells accumulate more DHA and also accumulate other intermediates, e.g., eicosatetraenoic acid (ETA, C20:4 n-3), EPA and DPA. On the other hand, undifferentiated WAT cells accumulate EPA, whereas differentiated WAT cells accumulate DPA instead of DHA [57]. Fatty acid desaturases are key enzymes in the remodeling of fatty acids, introducing a double bond at the Δ 5, Δ 6 or Δ 9 carbon of the fatty acid chain. These desaturases are Δ 5 desaturase (D5D or FADS1, fatty acid desaturase 1), Δ 6 desaturase (D6D or FADS2, fatty acid desaturase 2) and Δ 9 desaturase (D9D) (Figure 2) [58]. Elevated ALA concentration in adipose tissues is associated with lower prevalence of metabolic syndrome, and D6D may affect the conversion of ALA to EPA [59].

Resolvins and protectins are newly-discovered mediators that are enzymatically generated from n-3 fatty acid precursors [60]. The E-series resolvins (e.g., RvE1, RvE2 and RvE3) are produced from EPA, and DHA is a precursor of three distinct specialized pro-resolving lipid mediators (SPM) that include the D-series resolvins, protectins and maresins [61]. The lipoxins are generated from arachidonic acid (C20:4 n-6). The SPM are generated following n-3 PUFA supplementation [62] and also in transgenic mice overexpressing the *fat-1* (ω-3 fatty acid desaturase fat-1) gene that converts n-6 to n-3 fatty acids [63]. The low SPM concentrations in obese adipose tissues may result from n-3 PUFA deficiency, thus decreasing substrates for SPM biosynthesis [64].

## 5. The Beneficial Physiological Effects of n-3 PUFA

The type and quantity of fats in the diet are important factors in determining the risk for obesity [65]. The correlation of dietary fatty acid intake is stronger with concentrations in plasma than in erythrocytes [66], and the plasma n-3 PUFA are negatively correlated with metabolic syndrome [67,68]. Conversely, higher plasma levels of n-3 PUFA are associated with reduced obesity risk [69]. ALA conversion to longer chain n-3 PUFA, such as EPA and possibly DHA, may provide cardioprotection [70]. Hence, increasing n-3 PUFA in the circulation via dietary supplementation to reduce plasma TAG can lower the incidence of the obesity-induced metabolic syndrome, including insulin resistance, hypertension and dyslipidemia. Moreover, dietary PUFA supplementation along with a hypocaloric diet increases tissue PUFA (EPA and DHA) and the rates of ketogenesis and fatty acid oxidation [71].

### 5.1. The Beneficial Effect of n-3 PUFA in Human Studies

The effects of n-3 fatty acids on humans can start as early as embryonic development. First, the effects during the prenatal period indicate that maternal nutritional status may influence the fetus or the neonate to modulate the incidence of obesity and obesity-induced metabolic syndrome later in life; Secondly, dietary n-3 PUFA has been shown to be beneficial in children, as well, suggesting that these fatty acids can modulate health and adipose tissue development in the early postnatal years; Lastly, n-3 PUFA supplementation improves health outcomes and decreases obesity risk in adults, reducing the risk of obesity-related metabolic syndrome. The results of selected human trials regarding n-3 effects are summarized in Table 1.

#### 5.1.1. The Effects of n-3 PUFA in Different Periods

Human adipose tissues is detectable between the 14th and the 16th weeks of gestation with predominately multilocular fat cells, and fat depots are found at the beginning of the third trimester gestation [79]. Thus, adipose tissue development during this stage presents a unique opportunity for regulation by n-3 fatty acids. Unfortunately, there has been an increase in the dietary n-6/n-3 ratio in the diets of people in industrialized countries, and this affects the fatty acid composition of maternal plasma, adipose tissue and breast milk in lactating women. These compositional changes will alter fetal and neonatal adipogenic, lipogenic and adipokine gene expression in adipose tissues, which may potentially lead to the development of obesity later in life [80]. Obesity in children is increasing in prevalence worldwide [81], and the modulation of fat intake and its composition in the early-life period may prevent childhood obesity [82]. A large cohort study shows increased fat cell numbers and size starting from age three [83]. Modulation of fat intake and its composition in early life may prevent childhood obesity [82]. Higher n-3 PUFA concentrations in the maternal diet and in the umbilical cord plasma phospholipids are associated with lower adiposity in three-year-old children. Greater plasma arachidonic acid concentration is associated with increased subscapular and triceps skin fold thickness, BMI *z* score and plasma leptin concentrations [7]. Total lipid contents are significantly lower in the placentas of overweight and obese women supplemented with n-3 PUFA compared with a placebo group during mid- to late pregnancy (14.14 ± 1.03 vs. 19.63 ± 1.45 mg lipid/g tissue). n-3 PUFA inhibits placental lipid esterification pathways and reduces placental lipid accumulation [84]. Hence, reduced risk of childhood obesity is associated with enhanced maternal-fetal-neonatal n-3 PUFA.

A number of systematic reviews indicate that serum TAG is reduced 20%–30% by consuming n-3 PUFA derived from marine sources. The TAG lowering effects are variable depending on the daily intake of DHA [73]. When ≥4 g/day of n-3 PUFA is consumed, there is a 9%–26% reduction in circulating TAG, while a 4%–5% reduction occurs when 1–5 g/day of EPA and/or DHA are consumed [85]. In healthy male adults, fish oil or DHA-oil supplementation for 15 weeks lowers fasting plasma TAG concentrations and increases the plasma HDL2/HDL3 cholesterol ratio [72]; HDL3 content is associated with increased visceral fat deposition and impaired VLDL function [86]. Another study shows that plasma TAG is reduced about 25%–50% after one month of treatment with n-3 PUFA (3.4 g/day) [87]. Ingestion of the marine fatty acids, EPA and DHA in the form of ethyl esters for 12 weeks in 45 healthy normal-TAG males indicates that serum TAG and HDL3-cholesterol concentrations are reduced in a dose-dependent manner. However, VLDL and low density lipoproteins and total high density lipoprotein-cholesterol concentrations are not reduced [73]. These results indicate that n-3 PUFA, especially DHA, can lower plasma TAG in healthy subjects. Moreover, n-3 PUFA not only improves the hyperlipidemia in healthy populations, but also ameliorates dyslipidemia in overweight and obese individuals [75]. High EPA and DHA intakes also attenuate the association of obesity with dyslipidemia and low-grade systemic inflammation [88]. The intake of 0.3–3.0 g n-3 PUFA/day reduces body weight, body fats and metabolic syndrome in overweight, obese individuals and metabolic syndrome [30,76]. Replacement of neutral fats in the diet with fish oils reduces body fat mass (fish oil vs. control = −0.88 ± 0.16 vs. −0.3 ± 0.34 kg) [89]. Thus, n-3 PUFA attenuates the characteristics of metabolic syndrome, such as lowering of plasma TAG and body fat mass.

#### 5.1.2. The Effects of n-3 PUFA on Inflammation Factors in Humans

Obesity is associated with the presence of low-grade inflammation in WAT, which is characterized by macrophage infiltration and leads to production and secretion of inflammatory cytokines in the tissues. Inflammation increases the production of pro-inflammatory lipids and peptides, such as leukotriene (LTC4), interleukin 6 (IL-6) and tumor necrosis factor-α (TNF-α), by adipose tissue macrophages [90,91]. Production of anti-inflammatory molecules, such as adiponectin, is reduced [92]. Plasma markers of inflammation, such as IL-6, C-reactive protein (CRP) and, in subcutaneous adipose tissues, IL-6, monocyte chemoattractant protein-1 (MCP-1) and cluster of differentiation 68 (CD68), are increased in obese compared with lean adults.

In men, during a four-year follow-up period, n-3 PUFA intake is inversely associated with risk for metabolic syndrome, and high n-3 PUFA consumption significantly lowers the risk. On the other hand, there is no association between the incidence of metabolic syndrome and the intake of fish and n-3 PUFA in women [93]. The result shows the potential sex difference in the beneficial effect from n-3 PUFA supplementation. A combination of weight loss and n-3 PUFA supplementation reduces markers of metabolic syndrome without affecting the levels of inflammatory markers [77,78]. In moderately obese men, MCP-1, but not CRP, is lowered after fish oil supplementation (1.1 g/day EPA + DHA) for six weeks [94]. However, in the hyper-TAG men, who received 3 g/day DHA or 456 mg EPA + 375 mg DHA for 90 days [74], the plasma serum amyloid A (SAA) concentration is not altered [95]. Taken together, these results suggest that certain specific inflammatory markers are affected by n-3 PUFA supplementation. Even though the inflammatory markers may be affected by n-3 PUFA, a 5% weight loss does not decrease the plasma concentration of circulating inflammatory markers (IL-6, MCP1, CRP or white blood cell count) [36]. Although lipid flux, lipid synthesis, extracellular matrix remodeling and oxidative stress are markedly downregulated by a 5% weight loss, expression of inflammation markers is not downregulated until the weight loss reaches 16% [36]. The results indicate the limitations of n-3 PUFA to lower the level of inflammatory markers.

Other than the inflammatory markers, effects on the SPM also contribute to the anti-inflammation effects of n-3 fatty acids. For instance, in healthy volunteers, after supplementation of n-3 PUFA for seven days, a series of SPM, such as RvE1, RvD1, 18R/S hydroxyeicosapentaenoic acid (18R/S-HEPE), 17R/S (hydroxydocosahexaenoic acid (17R/S-HDHA) and 14R/S-hydroxydocosahexaenoic acid (14R/S HDHA), is increased [96]. The biologically-active SPM may contribute to the benefits of n-3 PUFA in metabolic syndrome. In contrast, in patients with elevated TAG levels supplemented with n-3 PUFA for 10 weeks, no RvE1, RvD1 or 17S-HDHA increase is detected [97]. These divergent findings suggest that insufficient SPM biosynthesis from n-3 PUFA in obesity or diabetes may limit the beneficial effect of n-3 fatty acids in reversing the deleterious effect of metabolic syndrome. However, larger scale population and mechanistic studies may be required to uncover the beneficial effects of SPM generated from n-3 PUFA [98].

#### 5.1.3. The Receptor for n-3 PUFA

Several fatty acids act as ligands for G-protein-coupled receptors (GPCRs) to activate intracellular signaling and exert physiological functions. G-protein coupled receptor 120 (GPR120) is a newly-found DHA receptor [99]. Adipose tissue expression of GPR120 is higher in obese than in lean subjects [100]. The sequence of the GPR120 exon in obese subjects reveals a deleterious non-synonymous mutation, which may attenuate beneficial n-3 PUFA effects and the secretion of glucagon-like peptide 1, leading to obesity [101]. Furthermore, the expression of the fatty acid binding protein 4 in omental adipose tissue is lower in the mutant carriers than in wild-type individuals [100]. The lack of fatty acid binding protein may lead to decreased availability of n-3 PUFA to act as ligands for peroxisome proliferator activated receptor γ (PPARγ) activation and, consequently, reduce the ability for insulin sensitization via PPARγ activation [102].

#### 5.1.4. Confounding Factors May Mask the Effect of n-3 PUFA in Human Studies

There are many confounding factors and limitations in human studies, which may mask the effects of n-3 fatty acids including the dose and source of n-3 PUFA, amounts of other fatty acids in the n-3 PUFA source, participant selection, genetic backgrounds, living conditions, general daily diet quality and quantity, etc. Another potential confounding factor, benign obesity, defined as metabolically healthy obese individuals (MHO) that are insulin sensitive with lower levels of ectopic fat in liver and skeletal muscle [103], should also be considered. The MHO group has lower mean omental adipocyte size (80.9 ± 10.9 μm) compared with metabolically unhealthy patients (100.0 ± 7.6 μm). Both subcutaneous and omental adipocyte sizes correlate positively with the degree of fatty livers, but only omental adipocyte size is related to metabolic syndrome [104]. Moreover, variation in a rate-limiting gene for PUFA synthesis, Δ 6-desaturase, can affect the prevalence of metabolic syndrome [59]. The lack of a consistent study design and variations in human responsiveness may be major limitations to assess the effects of n-3 PUFA and to establish a recommended dose for n-3 PUFA intake [85]. Hence, animal models in vivo and in vitro are required to reveal potential mechanisms for the n-3 PUFA effects.

### 5.2. The Benefits of n-3 PUFA in Animal Studies

Many functions of n-3 PUFA are still unknown, and comprehensive knowledge of the beneficial effects associated with n-3 PUFA remain to be discerned. Therefore, animal studies that allow careful consideration of species differences, genetics and individual metabolic variations serve as useful experimental models to ensure the reliability and reproducibility of data to suggest detailed mechanisms of n-3 PUFA effects in humans.

#### 5.2.1. The Effect of Endogenous n-3 PUFA in Animals

A transgenic animal model is used to investigate the conversion of endogenous n-3 PUFA. Transgenic mice overexpressing *fat-1* (ω-3 fatty acid desaturase derived from *C. elegans*) and fed a high n-6 unsaturated fat diet have a 23% reduction in the ratio of n-6/n-3 fatty acids, a 61% increase in EPA and a 19% increase in DHA levels in adipose tissues [105]. Although body weight is not significantly modified after a high-carbohydrate diet, a metabolite of n-3 PUFA, protectin D1, which is lacking in muscle and adipose tissue of high-fat-fed wild-type mice, is increased in high-fat-fed *fat-1* mice. These results indicate that restoration of n-3 PUFA prevents obesity-linked inflammation and insulin resistance [106]. Thus, endogenous n-3 PUFA is beneficial for coping with the effects of excessive caloric consumption.

#### 5.2.2. The Effect of Exogenous n-3 PUFA in Animals

Exogenous dietary n-3 PUFA changes the lipid profile of various tissues and the plasma in mammals, including rodents and pigs [107,108,109,110]. Prenatal fatty acid status also alters fetal and neonatal adipogenesis in humans [7]. The effect of maternal diets that increase n-3 PUFA supply to the offspring should be elucidated. Most information related to the plasma PUFA profile and adiposity at younger ages is limited in animal studies. In female rats fed DHA throughout pregnancy and lactation and with all pups weaned to a commercial rat chow, the total percentage body fat is increased in both male and female offspring of DHA-fed dams compared to controls, and the fat accumulation is primarily in subcutaneous depots [111].

Modification of the lipid profile may also change the basal metabolism [112]. Dietary unsaturated fat increases lipolysis by increasing the affinity of β-adrenergic receptors for their agonists by increasing the membrane fluidity of adipose tissues [113]. The n-3 PUFAs play a beneficial role in reducing adiposity and therefore to lower the incidence of the obesity-induced metabolic syndrome [112]. For example, in rats fed a diet with a high concentration of either saturated fatty acids (coconut oil or beef tallow) or polyunsaturated fatty acids (safflower oil, rich in n-6 PUFA), the adipose tissue norepinephrine-stimulated lipolytic rate is lower with the saturated compared to the unsaturated fat diet [114]. In addition, cAMP accumulation is lower in the saturated fat diet group leading to inhibition of downstream enzymes, such as cAMP phosphodiesterase and hormone-sensitive lipase. Furthermore, fish oil treatments or dietary EPA + DHA elevate gene expression for fatty acid β-oxidation and mitochondrial biogenesis in epididymal and subcutaneous WAT [115,116]. In addition, expression of PGC1α, nuclear respiratory factor1 (NRF1) and CPT1α genes is up-regulated without a change in UCP1 gene expression [114]. Another study shows that replacement of only 15% of dietary lipids with a mix of EPA and DHA stimulates the expression of the mitochondrial oxidative machinery and promotes β-oxidation of fatty acids in WAT of mice [107]. Thus, n-3 PUFA regulate the lipid and energy metabolism in WAT.

The distribution of adipose tissue may be affected by n-3 PUFA. A gain in fat mass is observed in all WAT (epididymal, retroperitoneal, mesenteric and subcutaneous) when mammals are fed with a high-fat diet, but only two abdominal WAT depots (epididymal and retroperitoneal) are specifically reduced by n-3 PUFA. Plasma TAG levels are positively correlated with retroperitoneal, epididymal fat cell size, and TAG levels are lowered with n-3 fatty acid supplementation [117]. Although, adipocyte number is not reduced by n-3 PUFA, the volume of adipocytes is significantly decreased in each adipose depot [118]. 

#### 5.2.3. Potential Mechanisms Mediate n-3 PUFA Effects

The n-3 PUFA are potential ligands for PPARγ, with a relatively weaker affinity compared to pharmacological agonists, such as glitazone [48]. Several fatty acid metabolites activate PPARγ better than the fatty acid per se [119]. n-3 PUFA also serve as substrates for the biosynthesis of SPM, such as resolvins and protectins, that may alter adipose tissue function and protect the liver from insulin resistance and hepatic steatosis in murine models of obesity [120]. Defective SPM biosynthesis from n-3 PUFA caused by adipose tissue inflammation [121] can be rescued by administration of exogenous SPM [64]. n-6 LA attenuates the cardio-protective action of resolvin D1 [122]. These results suggest that the increased n-6/n-3 ratio in modern diets may reduce beneficial SPM generated from n-3 PUFA and increase the incidence of obesity-induced metabolic syndrome. Taken together, n-3 PUFA reduce plasma TAG, suppress adipose tissue inflammation and enhance adipose fatty acid β-oxidation to reduce lipotoxicity in non-adipose organs [123].

#### 5.2.4. Suitable Animal Models for Dietary Intervention

To establish an animal model for studying the effects of a nutrient moiety, the metabolic rate and energy expenditure should be considered because these are affected by the size of an animal. For example, smaller mammals, such as mice, have a higher ratio of surface area to volume. Therefore, a larger relative heat loss to the environment occurs in smaller animals. To maintain a stationary body temperature like humans despite rapid heat loss across the body surface, a small animal has a higher fatty acid oxidation in cells and respiration in cells, which result in higher metabolic rates [124]. Conjugated linoleic acids (CLA) have been reported to lower body fats mediated by enhancement of energy expenditure. Theoretical calculations based on metabolic body weight and rate indicate that there should be seven-times more reduction in body fat in mice than in humans [125]. However, the body fat-lowering effects of CLA are more pronounced in humans than in mice, suggesting that the beneficial effects of n-3 PUFA are different in mice and humans. Pigs, on the other hand, have many metabolic features that are similar to humans. Their cardiovascular system, organ sizes and body fat distribution are similar to humans [116,126,127]. In addition, the body weight of some miniature pigs is approximately 70 kg, which is similar to mature adult humans [126]. Thus, the pig can be a good research model for demonstrating the function of PUFA in metabolic diseases. Fish oil consumption decreases postprandial lipemia and increases plasma lipid clearance in mini-pigs [128]. Male pigs fed with different sources of fat, including hydrogenated animal fat, menhaden oil or tuna oil, have no change in insulin metabolism. Nevertheless, a positive correlation is found between insulin resistance and body fat in pigs [129], suggesting that adult male pigs can be a reliable model for insulin metabolism. Moreover, in mini-pigs fed large amounts of n-3 PUFA, insulin sensitivity is enhanced [130].

Most rodent studies feed a high-energy diet and try to ameliorate metabolic syndrome symptoms with supplements of n-3 PUFA. However, diets containing n-3 PUFA in rodent studies tend to involve unrealistically high levels of n-3 PUFA relative to the human consumption pattern. Consequently, we fed pigs diets with 2% tallow or DHA (as-fed basis) for 18 days, and the body weight was not affected by different dietary fats. Expression of sterol regulatory element-binding protein 1 (SREBP1c; a transcription factor associated with lipogenesis) is inhibited, and acyl CoA oxidase (ACOX; promoting fatty acid oxidation) expression is increased in liver by DHA treatment [133]. To exclude the confounding endocrine effects by other organs in vivo, a primary porcine adipocyte system has been established [134] to investigate DHA effects. The expression of SREBP1c is inversely correlated with DHA treatment, whereas ACOX is positively correlated with DHA concentrations. Another study also found that ACOX and PPARα mRNA are increased in the subcutaneous fat and liver by dietary DHA supplementation at 9400 mg and that plasma TAG is lower [135]. Taken together, DHA treatment increases peroxisomal fatty acid oxidation and inhibits liver lipogenic genes to lower the TAG in circulation. The animal studies are summarized in Table 2.

## 6. The Molecular Mechanisms by which PUFA Affects Lipid Metabolism

**Figure 3:**
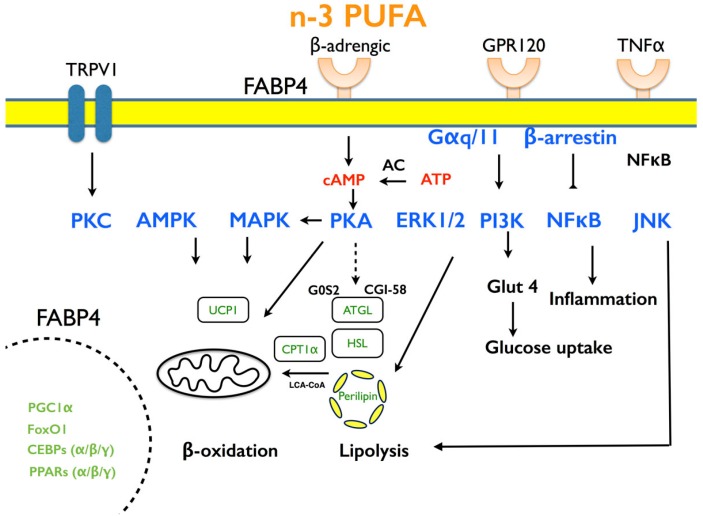
The mechanism of n-3 PUFA at the cellular level. TRPV1: transient receptor potential vanillin 1; FABP4, fatty acid binding protein 4; GPR120, G-protein coupled receptor 120; TNFα, tumor necrosis factor-α; PKC, protein kinase C; AMPK, 5’ AMP-activated protein kinase; MAPK, mitogen-activated protein kinase; PKA, protein kinase A; ERK1/2, extracellular signal-regulated kinase 1/2; PI3K, phosphoinositide-3 kinase; NFκB, nuclear factor κB; JNK, c-Jun N-terminal kinase; UCP1, uncoupling protein 1; CPT1α, carnitine palmitoyltransferease-1α; ATGL, adipose triglyceride lipase; HSL, hormone sensitive lipase; Glut4, glucose transporter 4; PGC1α, peroxisome proliferator activated receptor gamma coactivator 1-α; Foxo1, forehead box O 1; C/EBPs, CCAAT element binding proteins; PPARs, peroxisome proliferator activated receptors.

To understand the mechanisms for the effects of n-3 PUFA on lipid metabolism, adipocyte cell culture and clonal murine cell lines have been used. Compared to most rodent models, adipose tissue depots in pigs are of sufficient size for stromal vascular or adipocyte preparation without pooling across depots or animals [116,134]. These systems in vitro are effective for studying the PUFA functions in lipid metabolism. PUFA regulates the functions of adipocytes through modulation of activity and transcription factors that act as nutrient mediators [119]. Herein, the molecular mechanisms related to differentiation, lipid metabolism, energy expenditure and inflammation at the cellular level are discussed (Figure 3).

### 6.1. Adipogenesis

Adipogenesis occurs in two phases, determination and terminal differentiation [136]. It is regulated by complex and highly orchestrated gene expression programs [137]. Adipogenesis involves the differentiation of preadipocytes to mature adipocytes and is sensitive to nutrient concentrations. Understanding the processes that lead to de novo differentiation of adipocytes and the progression to obesity is necessary [119]. Individual n-3 PUFA may function as agonists or antagonists for several transcription factors related to adipocyte differentiation and function, including peroxisome proliferator-activated receptors (PPARs, α, β and γ) and liver X receptors (LXRs) [119]. Three classes of transcription factors, including CCAAT-enhancer-binding proteins (C/EBPs), PPARγ and the basic helix-loop-helix family (adipocyte determination- and differentiation-dependent factor 1, ADD1/sterol regulatory element-binding protein 1, SREBP1c) are all involved in adipogenesis [138]. Even though optimal affinity is achieved across all compounds containing 16–20 carbons, DHA is a better activator of PPARα than other fatty acids [139]. Besides, when porcine PPARγ is expressed ectopically in myoblasts, DHA serves as an effective ligand and increases the expression of the PPARγ targets, adipocyte protein 2 (aP2) and adiponectin [131]. Both adipogenic genes and glucose metabolism genes are elevated in PPARγ transgenic mice by dietary n-3 PUFA supplementation. These results indicate that DHA acts as a ligand for PPARα and PPARγ.

### 6.2. Lipid Accumulation

Exogenous fatty acids are stored as TAG that is sequestered into lipid droplets in adipocytes. This is the major pathway in adipocytes to regulate hypertrophy and, thus, the cell size of adipocytes [140,141]. Additionally, maintenance of lipid droplet size is regulated by several lipolytic enzymes, including adipose triglyceride lipase (ATGL) and hormone sensitive lipase (HSL) [142]. These two enzymes interact with perilipin and caveolin-1 within the lipid raft membrane of lipid droplets and depletion of functional perilipin and caveolin-1 increases lipolysis [143]. Protein kinase A increases lipolysis by phosphorylating two key substrates, perilipin (to inactivate) and HSL (to activate) [144]. Studies to discern the mechanisms for the effects of fatty acids on lipolysis in adipocytes present mixed results. During differentiation of 3T3-L1 cells into adipocytes, cells treated with 100 μM of EPA, DHA or DPA for seven days have decreased lipid accumulation with n-3 PUFA treatments, and medium glycerol, an indicator for lipolysis, is increased by DHA compared with the other fatty acids. Expression of three proteins related to lipid droplet formation (perilipin, caveolin-1 and cell death-inducing DFFA-like effector a, Cidea) is inhibited by DHA [145]. In contrast, another study indicates that incubation in vitro with 50 μM DHA in 3T3-L1 cells increases TAG accumulation and elevates the expression of the adipogenic-related genes, such as C/EBPα, PPARγ and aP2 [146]. The main difference between these two studies, is not only the concentration of fatty acid used (50 vs. 100 μM), but also the exact experimental protocol. In the first study, 3T3-L1 cells are differentiated for two days, then exposed to fatty acid as differentiation continued. However, in the second study, 3T3-L1 cells are pretreated with fatty acid for 24 h, then are induced to differentiate. Hence, even though the cell lines are the same, the timing of exposure to the PUFA is very different. In preadipocytes, DHA inhibits differentiation-associated mitotic clonal expansion, and post-confluent preadipocytes have increased apoptosis after DHA treatment for 48 h. Furthermore, DHA increases basal lipolysis in fully-differentiated 3T3-L1 cells after a four-hour incubation. The results suggest that DHA may exert is anti-obesity effect by inducing apoptosis in post-confluent preadipocytes [147]. To compare results in these in vitro experiments, the concentration of n-3 PUFA and the exact protocol, including the timing of the treatment, must be the same. Regardless, n-3 PUFA and particularly DHA strongly inhibit preadipocyte differentiation in vitro. The experiments in vitro should be designed to mimic a physiological state in animals, such as the prevention of adipocyte differentiation in preadipocyte [119] or ameliorating the expanded cell size in differentiated adipocytes.

Adipose-derived-related protein (ADRP, adipophilin as a human ortholog) [148] is a lipid droplet-associated protein, which regulates fatty acid uptake and storage. Expression of ADRP is regulated by retinoid x receptor (RXR) [149] and PPARγ via the PPAR response element (PPRE) [150]. The expression of ADRP is only modestly regulated by PPAR (α, δ and γ) agonists, and PPARδ is the most effective of these transcription factors [151]. Long chain PUFA, such as AA, DHA and EPA, stimulate ADRP mRNA expression to a greater extent than palmitic acid (PA). The expression of ADRP did not increase with the differentiation state. Furthermore, synthetic PPAR and RXR agonists only increase the gene expression of ADRP, but not protein levels. Nevertheless, DHA, AA and EPA induce the incorporation of oleic acid into TAG coinciding with the expression of ADRP mRNA and protein [151]. These results indicate that ADRP plays an important role in long chain PUFA uptake. Human [7] and rodent studies [111] show that maternal nutrition affects fetal development. Therefore, regulation of ADRP by different fatty acids during fetal development could be a way to regulate the uptake and utilization of fatty acids in the placenta.

Before GPR120 was identified as the receptor for DHA [99], GPR120 was known to be activated by long-chain fatty acids, and its presence was found in all adipose tissues (subcutaneous, perirenal, mesenteric and epididymal), pituitary gland, lung, small intestine, colon and spleen. The expression of GRP120 expression is greater in mature adipocytes than in stromal-vascular cells (preadipocytes) [152], and expression of adipogenic genes and lipid accumulation are suppressed with knockdown and gene deficiency of GPR120, indicating the important role of GPR120 in adipocyte differentiation and maturation [153]. In mature adipocytes and 3T3-L1 adipocytes, GPR120 increases glucose transport and translocation of GLUT4 to the plasma membrane [99]. Mice deficient in GPR120 develop obesity, glucose intolerance and fatty livers, suggesting that GPR120 plays a role in lipid metabolism.

### 6.3. Energy Expenditure

Long-chain fatty acids constitute the bulk of dietary fats, which makes them the principle fatty acids stored in adipose TAG and, thus, a predominant source of energy under normal conditions and the major product of fasting-induced lipolysis. To reduce body fat storage, diet-derived or stored TAGs need to be hydrolyzed into fatty acids and mobilized for energy usage via oxidation [23]. Fatty acid β-oxidation occurs both in mitochondria and peroxisomes [154]. The β-oxidation in mitochondria produces more energy than in peroxisomes [155]. Fatty acids are completely oxidized to acetyl-CoA by mitochondrial β-oxidation, and carnitine palmitoyltransferase 1α (CPT1α) plays an important role as the rate-limiting enzyme [156]. In 3T3-L1 adipocytes incubated with EPA, the CPT1α activity is increased by altering the structure and dynamics of the mitochondrial membranes [157]. Peroxisomal ACOX catalyzes the first and rate-limiting enzymatic step of the peroxisomal fatty acid β-oxidation pathway [158].

Fish oil increases UCP1 expression in adipose tissue [107,159]. However, the mechanism is still unclear. Recently, an explanation for browning of adipose tissues by fish oil has been shown to be via the sympathetic nervous system activation [44]. Supplementation with dietary fish oils increases oxygen consumption and rectal temperature in C58BL/6 mice, indicating that energy expenditure is increased. In addition, markers of beige adipocytes, UCP1 and β3 adrenergic receptor (β3AR) are both up-regulated in inguinal WAT [44]. To elucidate the energy expenditure functions of DHA, knockout mice for the transient receptor potential cation channel subfamily V member 1 (TRPV1) are used as a model. TRPV1 is an ion channel with high calcium permeability, which is expressed in nociceptive neurons, brain, gastrointestinal tract and adipocytes [160,161,162]. Compared with EPA and ALA, DHA exhibits significantly greater efficacy to active TRPV1 in a protein kinase C-dependent manner [163]. Energy expenditure is not increased in the TRPV1 knockout mice, suggesting a role for this protein. DHA may induce the systematic nervous system to activate TRPV1 activity and expression in the afferent nerves of the gastrointestinal tract, leading to an up-regulation of UCP1 in interscapular BAT and inguinal WAT. The effect of DHA on energy expenditure appears to be a secondary effect and not a direct effect on adipose tissue. Regardless, the possibility that TRPV1 is affected by DHA in adipose tissues warrants serious consideration. Compared with obese rodents or humans, the expression of TRPV1 in lean counterparts is greater in visceral adipose tissues, indicating the expression of TRPV1 in adipocytes may play a role in preventing adipogenesis and obesity. Obese human male subjects have reduced TRPV1 expression [160]. Therefore, these observations suggest that obesity may impair TRPV1 functions or expression, and DHA may prevent obesity and metabolic syndrome partly through restoring TRPV1 function.

In addition to the ion channel receptor TRPV1 and GPR120, another potential mediator of DHA effects could be the major facilitator superfamily domain-containing protein 2a (Mfsd2a), which was an orphan transporter later defined as a DHA transporter involved in normal brain growth and cognitive function [164]. It is expressed in many tissues and is highly induced in liver and BAT during fasting. Furthermore, Mfsd2a is induced during cold-induced thermogenesis, suggesting a function in adaptive thermogenesis [165]. The expression of Mfsd2a is induced by dibutyryl-cAMP or cAMP-inducing agents, which suggest that the regulation is mediated by β-adrenergic signaling. The potential of DHA to stimulate BAT energy expenditure mediated through Msfd2a needs to be elucidated.

### 6.4. Inflammation

DHA attenuates the secretion of proinflammatory adipokines, such as chemerin, IL-6 and MCP-1, in human adipocytes in vitro [166]. In rodents in vivo, gavage with DHA causes inhibition of gene expression of proinflammatory cytokines, such as TNFα, IL-1β, MCP-1 and IL-6, in liver [132]. Even though adipocytes were initially thought to be the cellular source of proinflammatory mediators in obesity, it was later established that infiltrated macrophages in obese fat are the major source of exacerbated production of inflammatory mediators [167]. Chemotaxis of macrophages is decreased by an n-3 PUFA diet, and the expression of inflammatory genes, such as IL-6, TNF-α, MCP-1, IL-1β, iNOS (inducible nitric oxide synthase) and CD11c, is reduced. The n-3 PUFA not only inhibits the expression of proinflammatory factors, which cause low-grade inflammation in adipocytes, but may produce secondary effects in macrophages and monocytes (macrophage precursors). Note that SPM effects are discussed in the animal study section.

## 7. The Interplay between Liver and Adipose Tissues

Most studies focus on the effects of n-3 PUFA in a single organ, such as the liver, adipose tissue or muscle, etc. However, the interactions between organs should also be considered. In spite of limited information, the effect of DHA on the liver-adipose axis has been demonstrated [168]. Herein, the mechanisms of n-3 PUFA in liver and also the effect of hepatokines on adipocytes will be discussed. There is rapid turnover of accumulated EPA, DPA and DHA in the liver, a slower loss from omental fat and muscle TAG pools and an even slower loss from epididymal adipose tissues [9]. The GPR120-deficient mice with a dysfunction in lipid metabolism have TAG accumulation in the liver after feeding on a high-fat diet [100]. This result not only shows the importance of the DHA receptor in adipose tissues, but also addresses how adipocyte lipid metabolism causes major changes in the liver. Down-regulating the activity of PPARs, SREBPs and insulin receptor substrate 2 and up-regulating AMP-activated protein kinase in the liver improve glucose utilization and increase insulin sensitivity [169]. PPARα is mainly expressed in tissues with a high capacity for fatty acid oxidation, such as the liver, heart and skeletal muscle. In rodents, dietary PUFA induces the expression of PPARα target genes in the liver to stimulate fatty acid β-oxidation and reduce TAG [170]. At least in rodents, PUFA-induced lipid oxidation (through activation of PPARα) is clearly more relevant for the liver than for WAT [27]. When the human V162 allele and L162 variants of PPARα are transfected into HepG2 cells that are trans-activated with n-3 PUFA, the LPL activity is elevated in L162-PPARα cells [171]. Consumption of n-3 PUFA increases fatty acid β-oxidation in the liver through activation of CPT1α [172,173]. SREBP1c is a transcription factor that regulates the expression of lipid synthesizing enzymes involved in fatty acid and TAG accumulation and is a primary hepatic activator of lipogenesis [174]. Fish oil feeding inhibits the activation of SREBP1c by proteolysis. Moreover, the expression of PPARα and its targets, such as ACOX, medium-chain acyl-coenzyme A dehydrogenase, acyl-CoA synthase, LPL and UCP2, in the liver is not changed by fish oil [175]. Finally, our group showed that DHA negatively regulates forehead box O (FoxO) in both the liver and adipose tissue to decrease TAG synthesis and VLDL assembly to lower the plasma TAG [176].

### 7.1. Lipotoxicity in Livers Regulated by n-3 PUFA

Excessive dietary fats in circulation cause lipotoxicity in non-adipose organs, especially muscle and liver. Overflow of FFA into the liver leads to unconstrained lipid accumulation, which further contributes to the pathogenesis of non-alcoholic fatty liver and non-alcoholic steatohepatitis [177]. Saturated fatty acids (SFA), such as palmitic acid (C16:0) or stearic acid (C18:0), induce inflammation and lipotoxicity not only in macrophages, but also in metabolic tissues, such as the liver and adipose tissues [178,179]. SFA promotion of lipotoxicity in the liver is mainly mediated through enhanced endoplasmic reticulum stress and apoptosis. Conversely, n-3 PUFA (EPA or DHA) supplementation reverses SFA-induced hepatic inflammation and lipotoxicity [180,181]. For example, dietary n-3 PUFA consumption (2 g/day) for six months significantly improves human non-alcoholic fatty liver disease in subjects with decreased plasma alanine aminotransferase (ALT), TAG and TNF-α and homeostasis model assessment [182]. These protective effects of n-3 PUFA in liver can be partly attributed to the DHA-derived SPM, such as protectin D1 and 17S-hydroxy-DHA [164].

### 7.2. Hepatokines Regulated by n-3 PUFA

#### 7.2.1. Serum Amyloid A

Serum amyloid A (SAA) is an apolipoprotein mainly synthesized in mammalian liver. The acute-phase SAA is induced by IL-1, TNF-α and IL-6. In addition, SAA can replace apolipoprotein A1 as the major apolipoprotein of high density lipoprotein [165]. Serum SAA is correlated with the degree of obesity [183]. Human recombinant SAA inhibits the expression of lipogenesis-related genes and promotes the expression of lipolysis-related genes in hepatocytes. SAA also increases the lipolytic activity in human adipocytes. Treatment with the n-3 PUFA, DHA increases the SAA, IL-6 and TNFα expression and decreases perilipin expression in human adipocytes. [184]. We demonstrated that DHA promotes SAA expression mediated by the promoter region of C/EBPβ via protein kinase A activities [185]. Hence, we suggest that DHA could increase SAA expression and promote adipocytes lipolysis. Our study in pigs in vivo using suppression subtractive hybridization shows that hepatic SAA is upregulated by dietary DHA [186]. Furthermore, porcine SAA promotes lipolysis mediated by decreased expression of perilipin in pig adipocytes [187]. The mechanism of SAA-induced lipolysis is via extracellular signal-regulated kinase (ERK)/PPARγ and PKA signaling [188]. These beneficial effects mediated by DHA to alter lipid metabolism could be a new approach to cope with the incidence of obesity.

#### 7.2.2. Fibroblast Growth Factor 21

Physiologically, liver, but not adipose tissue, is the major source of secreted fibroblast growth factor 21 (FGF21) into circulation as an endocrine hormone. FGF21 is positively correlated with the severity and progression of non-alcoholic fatty liver disease [189]. FGF21 is induced in the livers by PPARα and in WAT by both a high fat diet and specific PPAR agonists [190]. The FGF21 plasma levels are independently correlated with hepatic fat contents and markers of hepatic apoptosis in obese youth [191]. Circulating FGF21 is decreased by supplementation with fish oil treatments [192]. Fish oil supplementation can lower the level of FGF21 in circulation, but the mechanisms are currently not clear.

#### 7.2.3. Angiopoietin-Like 4

The Angiopoietin-like 4 (ANGPT4) reduces the clearance of VLDL and chylomicrons by inhibition of LPL activity [193]. The ANGPT4 levels are positively correlated with fasting free fatty acids (FFA) and adipose lipolysis. The function of ANGPT4 may be protect tissues from FFA-induced cellular toxicity via a reduction in hydrolysis of TAG [194]. There is ubiquitous expression of ANGPT4 with high expression in WAT and the liver [195]. The mechanism for reducing circulating ANGPT4 is still unknown, but an increase in plasma free fatty acids by lipid infusion attenuates the reduction of ANGPT4 during a hyperinsulinemic euglycemic clamp [196]. The insulin-mediated decrease in plasma ANGPT4 is partially reduced during olive oil infusion and completely blunted during soybean oil and fish oil/medium chain TAG/long-chain TAG infusion [195]. There is no association between expression of ANGPT4 in WAT and LPL activity in WAT [197]. The expression of ANGPT4 is induced by DHA (C22:6 n-3) 70-fold compared with a 27-fold increase by linoleic acid (C18:2 n-6) and a 15-fold increase by oleic acid (C18:1) after a 6-h intravenous infusion [195]. The results suggest that ANGPT4 can be strongly regulated in the short term by DHA, and ANGPT4 may protect against the overflow of plasma FFA to cause lipid accumulation and lipotoxicity in non-adipose tissues.

## 8. Conclusions and Future Perspectives

Understanding mechanisms for n-3 PUFA’s effects are primarily derived from animal or cell studies because of the limitation for conducting such studies in human subjects/tissues. Species differences, as well as differences in experimental design must be factored into the interpretation of results from these studies. As a prevention strategy, the recommended human intake of EPA and DHA is between 0.5% and 2% of total energy intake [198]. Most human studies utilize a combination of DHA and EPA as an n-3 PUFA supplement, which may mask the effects of each individual fatty acid. The individual effect of EPA and DHA must be considered and the source of these compounds (marine or vegetable source) must be noted, as well, because the composition of the individual source is highly variable [24,199]. The plasma lipid profile plays an important role in metabolic syndrome incidence and severity [67,68,200]. Techniques for high-throughput and sensitive analysis of blood plasma lipids should be further developed [201]. Thus, routine plasma examination could be applied for monitoring health status.

To treat patients with obesity or its comorbidities, the amount of fish oils required to appropriately serve as ligands for the n-3 PUFA receptor GRP120 will be too high to be practical [85]. Long-term daily dietary n-3 PUFA consumption will be needed. Although n-3 PUFA also play a role as PPAR ligands, a synthetic PPARγ agonist reduces the symptoms of obesity, but also has many side effects, including induction of weight gain, fluid retention and bone fractures [202]. Alternatively, a high-affinity small molecule agonist for GPR120 has been identified and mitigates insulin resistance [203]. Furthermore, administration of n-3 PUFA-derived SPM mediators may resolve adipose tissue inflammation caused by obesity. For example, resolvin D1 exerts anti-inflammatory properties at nanomolar concentrations, whereas the precursor DHA is effective only at a micromolar level, suggesting that resolvin D1 may be more effective for ameliorating inflammation in obesity [204]. Moreover, deletion of D6D lowers the conversion of ALA to EPA [59]. Expression of D6D is regulated by PPARδ [205], suggesting that not only a GPR120 agonist, but a D6D inducer may lower the prevalence of metabolic syndrome. However, a gender difference must be considered because estrogen increases the D5D and D6D activity [206].

The goal of ameliorating the symptoms of metabolic syndrome to a large extent involves lowering ectopic fat deposition by decreasing hepatic lipogenesis/adipogenesis and to maintain healthy adipose functions. The interplay between adipose and liver tissues influenced by n-3 PUFA is an important contributor to control metabolic syndrome. However, n-3 PUFA also regulate lipid and glucose metabolism in muscles and pancreatic islets by preventing lipotoxicity and inflammation [207,208]. An effective control of metabolic syndrome will require a comprehensive understanding of lipid and carbohydrate metabolism not only in adipose tissue and liver, but also the interplay of metabolism in various organs.

## Figures and Tables

**Table 1:** Potential beneficial effects of dietary n-3 PUFA (EPA or DHA) supplementation in human studies.

Species	Treatment or Dosage	Duration	Observation	Reference
Mother-child pair	1. At 29 weeks gestation, the food-frequency questionnaire (FFQ) quantified the average frequency of consumption of >140 specified foods and beverage. 2. 0.15 ± 0.14 g DHA + EPA/day.	3 months	1. Maternal plasma DHA + EPA was 1.9% ± 0.6%, and umbilical plasma concentration was 4.6% ± 1.2%. 2. DHA + EPA = lower skin fold thickness and odds of obesity. 3. Maternal plasma DHA + EPA not associated with child adiposity.	[7]
Healthy men	1. Fish diet group: 4.3 ± 0.5 fish meals (provided 0.38 ± 0.04 g EPA and 0.67 ± 0.09 g) per week. 2. Fish oil group: oil 1.33 g EPA and 0.95 g DHA per day. 3. DHA-oil group: DHA-oil (EPA-free) 1.68 g DHA per day.	15 weeks	Fish, fish oil or DHA = lower plasma TAG concentration and total chylomicron+increased HDL2/HDL3 cholesterol.	[72]
Healthy men	0, 3, 6, or 12 capsules/day, (Each capsule provided 300 mg EPA and 200 mg DHA)	12 weeks	3 and 6 g n-3 fatty acids were similar, but VLDL, LDL and total HDL-cholesterol subtractions were no significantly different.	[73]
Moderately hyperlipidemic but otherwise healthy men	1. Placebo (7.5 g olive oil/day) 2. DHA capsules (7.5 g DHA oil/day)	90 days	1. The inflammatory markers was no difference within 45 days. 2. The CRP and IL-6 decreased, and anti-inflammatory matrix metalloproteinase-2 was increased at 90 days. 3. SAA positively associated with sum of saturated fatty acids.	[74]
Healthy weight, overweight and obese adults	2 g/day of algal DHA	4.5 months	1. DHA-supplemented group, the decrease in mean VLDL particle size and increase in LDL and HDL. 2. DHA supplementation reduced VLDL and total TAG.	[75]
Healthy weight, overweight and obese adults	1. >2 fatty fish meals/week 2. Restricted diet	Not mentioned	1. Plasma n-3 PUFA lower in obese participants. 2. Inversely correlated with EPA and DHA intakes.	[69]
Metabolic syndrome	1. Control 2. 1 g fish oil containing 180 mg EPA + 120 mg DHA	6 months	Reduced body weight and serum concentration of LDL-cholesterol, TAG in fish oil group.	[76]
Metabolic syndrome	1. Placebo = 4 or 6 g, soybean oil/day 2. Low flaxseed oil = 2.2 g ALA/day 3. High flaxseed oil = 6.6 g ALA/day 4. Low fish oil = 700 mg EPA + 500 mg DHA/day 5. High fish oil = 2.1 g EPA + 1.5 g DHA/day	8 weeks	1. The inflammatory marker were no significantly different. 2. Decreased plasma TAG and blood pressure decreased significantly+ increased LDL cholesterol.	[77]
Overweight hyperinsulinaemic women	1. Control = no weight loss + placebo oil 2. Weight loss program 10% weight loss + 5 g n-3 PUFA containing 1.3 g EPA + 2.9 DHA/day 3. Weight loss + placebo oil containing 2.8 g linoleic acid and 1.4 g oleic acid/day	24 weeks	1. Weight loss with both diets. 2. Diet n-3 PUFA increased adipose tissue n-3 PUFA. 3. Weight loss group improved insulin sensitivity. 4. n-3 PUFA increased plasma TAG and adipoinectin.	[78]

n-3 PUFA, n-3 polyunsaturated fatty acids; ALA, α-linolenic acid; EPA, eicosapentaenoic acid; DHA, docosahexaenoic acid; TAG, triacylglycerol; FFQ, food-frequency questionnaire; VLDL, very low density lipoprotein; LDL, low density lipoprotein ; HDL, high density lipoprotein; CRP, C-reactive protein ; IL-6, interleukin 6; SAA: serum amyloid A.

**Table 2:** Beneficial effects of n-3 PUFA in various animal models.

Species	Breed	Treatment	Duration	Observation	Reference
Mice	C57BL/6	1. Standard chow group 2. High-fat lard group 3. High-fat lard plus fish oil (FO) group (40 g soybean oil + 119 lard + 119 FO) 4. High fat fish oil group (40 g soybean oil + 238 g FO)	8 weeks	Fish oil decreased adipose tissue, body mass gain and insulin resistance even with lard.	[115]
Mice	Male C57BL/6J	1. 20% flax-seed oil 2. sHFf-F2 diet and 44% of lipids were replaced by n-3 PUFA concentrate (6% EPA and 51% DHA)	5 weeks	1. Fatty acid oxidation genes, Ppargc 1α, Nrf1 and Cpt1a elevated in epididymal fat by EPA + DHA. 2. Mitochondrial protein and induce β-oxidation in edidymal fat but not dorsolumbar fat in EPA + DHA.	[107]
Mice	Muscle specific-PPARγ transgenic mice	1. Control, 58% carbohydrate, 13.5% fat and 28.5% protein 2. Control + 4 mg Rosiglitazone/kg 3. High fish oil group containing (36% carbohydrate, 35.5% fat and 28.5% protein) 4. High-beef tallow diet (36% carbohydrate, 35.5% fat and 28.5% protein)	4 months	1. The PPARγ transgenic mice increased the expression of muscle Glut 4. 2. Fish oil group increased adipogenic and glucose uptake genes and lower blood glucose in transgenic mice. 3. Adiponectin elevated by fish oil.	[131]
Mice	Male C57BL/6 mice	**Treatment:** High fat diet induction for 23 weeks, then daily gavage with supplement. **Supplement:** 1. 0.5 or 1% DHA (based on the average daily dietary intake) 2. 1% DHA 3. 0.5% lysine + 1% DHA 4. 1% lysine + 1% DHA	4 weeks	1. The mRNA expression of hepatic pro inflammatory cytokines were suppressed by DHA and combinations of DHA + Lysine. 2. The lipogenic gene, ACC1 was suppressed by DHA. 3. Combination of DHA and lysine inhibited ACC1, fatty acid synthase, lipoprotein lease in gonadal adipose tissue. 4. The symptoms of nonalcoholic fatty liver disease were decreased by DHA and lysine.	[132]
Mice	1. Male C57BL/6J wild-type (WT) mice 2. Male BKS.Cg-Dock 7m+/+Leprdb/J (db/db) mice 3. Lean nondiabetic littermates (db/+)	**Treatment:** 1. 18 weeks of the HF diet (60% kcal from fat) or 2. low-fat (LF) control diet (10% kcal from fat) **Dose:** 1. DHA (4 µg/g body weight),17-HDHA (50 ng/g body weight) 2. Control (0.9% NaCl containing 3% delipidated fatty acid-free BSA and 2% ethanol)	Intraperitoneal injection every 12 h for 8 days or continuous application with osmotic (Alzet) pumps (∼120) for 15 days	1. Genetic and diet-induced obesity decreases adipose tissue n-3 PUFA–derived lipid mediators 17-HDHA and PD1. 2. Adipose tissue 17-HDHA and PD1 decreased after only 4 days of HF diet. 3. Greater adipose tissue 17-HDHA, which reduced adipose tissue inflammation. 4. Attenuated inflammation and improved insulin sensitivity induced by n-3 PUFA linked to increase SPMs and their precursors in adipose tissue. 5. Treatment with 17-HDHA reduces obesity-induced adipose tissue inflammation. 6. Treatment with 17-HDHA improves metabolic regulation in obesity.	[121]
Rat	Wistar rats	1. Control (sucrose replaced by starch) 2. Sucrose-rich (SRD) diet (corn oil, 8/100 g) 3. SRD + fish oil, fish oil 7/100 g + 1/100g corn oil	6 + 2 months	1. Fish oil decreased plasma TAG, VLDL and adipocyte size in sucrose diet. 2. Fish oil reversed dyslipidemia and improved insulin action and glucose sensitivity in muscle.	[112]
Rat	Female wistar Rat	**Treatment:** 1. Control (5% fat, 0.22% n-3 PUFA of total fatty acids) 2. 5% fat, 1.29% n-3 PUFA of total fatty acids **Dose:** 1. 6.5~9.0 mg n-3 PUFA/day	Dams = 3 weeks gestation + 3 weeks suckling; Offspring = 3 weeks post-weaning	1. n-3 PUFA increased body fat in males and females at 6 weeks of age. 2. Fat mostly subcutaneous depots, but no up-regulation of adipogenic/lipogenic gene transcription.	[111]
Rat	Sprague-dailey male rats	**Treatment:** 1. 14% coconut oil, beef fat or safflower oil 2. 900 mg/g 3. 10% n-3 PUFA	4 weeks	1. Norepinephrine-stimulated lipolysis was 50% lower in saturate diet. 2. The activities of 3’-5’-cyclic nucleotide (cAMP) phosphodiesterase and hormone sensitive lipase were lower in saturated fatty acids compared with polyunsaturated fatty acids.	[114]
Pig	Male castrated minipigs	**Treatment:** 1. Diet enriched with cod liver oil 2. Diet enriched with a mixture of sunflower palm and olive oil **Dose:** 1. ∼2.5 g/day (n-3) PUFA	4 weeks	1. The fish oil-enriched diet was associated with lower TAG, glycerol and nonesterified fatty acid concentrations in the hours after the gastric fat load than the control diet. 2. No significant effect of fish oil supplementation on plasma triacylglycerol clearance in minipigs.	[128]
Pig	Healthy duroc boars	**Dose:** 1. 62 g hydrogenated animals fat 2. 60 g menhaden oil containing 10.8 g DHA and 9.0 g EPA 3. 60 g tuna oil containing 19.8 g DHA and 3.9 g EPA	7 months	Long term supplementation of dietary n-3 PUFA did not affect insulin metabolism, but n-3 PUFA increase the fat accumulation.	[129]

sHFf, high fat diets with partial replacement of lipids by fish oil concentrate; FO, fish oil; SRD, sucrose-rich diet; cAMP, 3’-5’-cyclic nucleotide; n-3 PUFA, n-3 polyunsaturated fatty acid; DHA, docosahexaenoic acid; EPA, eicosapentaenoic acid; TAG, triacylglycerol; PPARγ, peroxisome proliferative activated receptor; Ppargc1α, PPARγ coactivator 1α; Nrf1, nuclear respiratory factor 1; CPT1α, carnitine palmitoyltransferase 1α; SPM, specialized proresolving lipid mediators; 17S-HDHA, 17-hydroxydocosahexaenoic acid; PD1, protectin D1.
